# Somatic Mutations of Hematopoietic Cells Are an Additional Mechanism of Body Aging, Conducive to Comorbidity and Increasing Chronification of Inflammation

**DOI:** 10.3390/biomedicines10040782

**Published:** 2022-03-27

**Authors:** Yegor E. Yegorov, Anastasia V. Poznyak, Evgeny E. Bezsonov, Alexander D. Zhuravlev, Nikita G. Nikiforov, Khava S. Vishnyakova, Alexander N. Orekhov

**Affiliations:** 1 Engelhardt Institute of Molecular Biology, Russian Academy of Sciences, 119991 Moscow, Russia; khava58@mail.ru; 2 Institute for Atherosclerosis Research, 121609 Moscow, Russia; 3 Laboratory of Angiopathology, Institute of General Pathology and Pathophysiology, 125315 Moscow, Russia; evgeny.bezsonov@gmail.com (E.E.B.); zhuravel17@yandex.ru (A.D.Z.); nikiforov.mipt@googlemail.com (N.G.N.); 4 Institute of Human Morphology, 117418 Moscow, Russia; 5 Department of Biology and General Genetics, I.M. Sechenov First Moscow State Medical University (Sechenov University), 105043 Moscow, Russia; 6 Center for Precision Genome Editing and Genetic Technologies for Biomedicine, Institute of Gene Biology, 119334 Moscow, Russia

**Keywords:** aging, somatic mutations, inflammation, CHIP, bone marrow, monocytes, chronification of inflammation, mitochondria

## Abstract

It is known that the development of foci of chronic inflammation usually accompanies body aging. In these foci, senescent cells appear with a pro-inflammatory phenotype that helps maintain inflammation. Their removal with the help of senolytics significantly improves the general condition of the body and, according to many indicators, contributes to rejuvenation. The cells of the immune system participate in the initiation, development, and resolution of inflammation. With age, the human body accumulates mutations, including the cells of the bone marrow, giving rise to the cells of the immune system. We assume that a number of such mutations formed with age can lead to the appearance of “naive” cells with an initially pro-inflammatory phenotype, the migration of which to preexisting foci of inflammation contributes not to the resolution of inflammation but its chronicity. One of such cell variants are monocytes carrying mitochondrial mutations, which may be responsible for comorbidity and deterioration in the prognosis of the course of pathologies associated with aging, such as atherosclerosis, arthritis, osteoporosis, and neurodegenerative diseases.

## 1. Introduction

Inflammation is an evolutionarily developed, very complex process aimed at restoring the status quo in the body. Any accidental changes to it should, in most cases, reduce its functionality and efficiency. If we believe that inflammation restores the structure and functions of the body, then a decrease in the efficiency of this process should slow down the recovery processes or do it poorly, leading to the development of chronic inflammation.

The key players in inflammation are immune cells. Among them, macrophages play a special role. Macrophages are involved in inflammatory processes in a variety of ways: they present antigens, they phagocytize objects with signs of foreign genetic information, as well as any cellular debris, they regulate the work of other cells, including both cells of the immune system and others (smooth muscle cells, endotheliocytes, cells of connective tissue). It is macrophages that complete inflammation, as they say, contribute to its resolution.

The precursors of macrophages, monocytes, are constantly formed during the work of the bone marrow, and then these cells spread throughout the body. In the case of an already existing inflammatory process, monocytes/macrophages migrate to the inflammation zone, begin to take part in it, and largely determine the fate of this inflammatory focus. We can consider one of the special cases, apparently quite common. A monocyte has a mitochondrial mutation that leads to mitochondrial instability. The destruction of mitochondria leads to the appearance of mitochondrial DNA in the cytoplasm of cells. Monocyte/macrophages are equipped with various receptors of innate immunity. PRRs (pattern recognition receptors) recognize mitochondrial DNA (which bears a resemblance to bacterial one) as a foreign object, and the cell begins to trigger inflammation exactly as if bacteria had entered the body. The macrophage starts to work as a sensor for a bacterial attack in its absence. When such a macrophage migrates to an area of pre-existing inflammation, the possibility that the inflammation will never be completed increases. It is important to introduce here two subpopulations of macrophages, M1 and M2. M1 macrophages, also called classically activated, respond to stimuli such as LPS, IFN-γ, and are important producers of pro-inflammatory cytokines. M2 macrophages are also called alternatively activated, respond to stimuli such as IL-4 or IL-13, and produce anti-inflammatory cytokines.

Such a mechanism should combine various inflammatory processes within one organism and have an effect similar to that of senescent cells. In the area of inflammation, changes in the extracellular matrix occur, the structure of tissues is disturbed, and their functionality decreases.

In recent years, many scientists have been working on senolytics—substances that selectively kill senescent cells. Senolytics can induce actual rejuvenation of the body, measured by various parameters. It is believed that the main mechanism of action of senolytics is to suppress inflammatory processes. Senescent cells acquire senescence-associated secretory phenotype (SASP) over time and, due to inflammation, accelerate and generalize aging processes. The corresponding term, inflammaging, has also appeared, which closely links the processes of inflammation and aging. Inflammation and telomere-dependent cell senescence can reinforce each other in accelerating aging [1,2].

Thus, if a certain mechanism contributes to the intensification of inflammatory processes in the body, we can assume that this mechanism accelerates aging. In our review, we will further consider more specific aspects of the influence of mutations in somatic cells of the bone marrow on the course of inflammatory processes.

## 2. Nuclear DNA Mutations in Hematopoietic Cells

With age, cells in our body accumulate somatic mutations, and somatic mosaicism develops in many tissues [3]. These mutations occur by chance and, therefore, in most cases they usually have no effect, or the effect is negative and in rare cases positive. In contrast to germline mutations, which can be eliminated by selection, somatic mutations in most cases are retained in tissues and are little subject to elimination. Based on this property, theories of aging were previously proposed based on the progressive accumulation of somatic mutations. A random change in the properties of cells within tissues should have a clear negative effect on the processes of normal development and maintenance of the body [4,5,6]. At the same time, few cases are known when the mechanism of the negative action of random mutations is directly described. Cancer patients are an exception. It should be noted that the rate of development of mosaicism is higher in rapidly dividing tissues, including bone marrow [7]. If the mutation gives the cells an advantage in growth, survival, etc., clones of altered cells, including cells of the immune system, can be formed.

**Figure 1:**
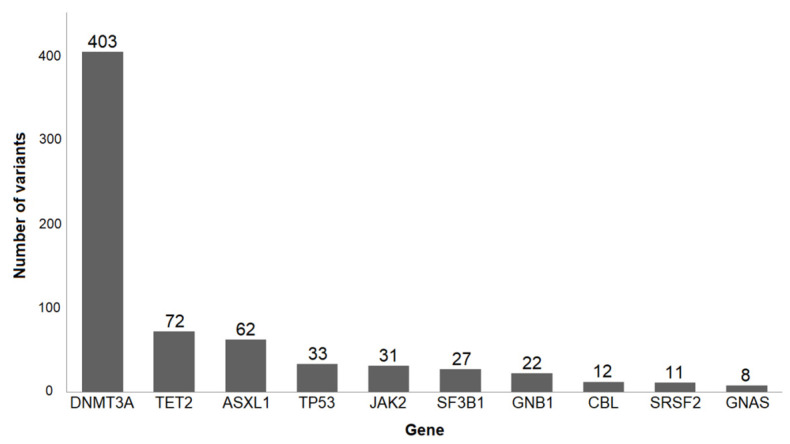
10 most frequently mutated genes implicated in hematologic cancers (modified from [11]). 693 samples were investigated.

The development of modern sequencing methods has made it possible to conduct very in-depth studies of the stability of the genome, including the possibility of working with individual cells. In the last decade, there have been a lot of works evaluating somatic mutations in blood cells. Initially, researchers were interested in the issues of carcinogenesis. Therefore, special attention was paid to genes, whose participation in carcinogenesis has been confirmed repeatedly. It turned out that somatic mutations occur more often than expected [8,9,10]. For example, in the article by Jaiswal et al. [11], peripheral blood whole-exome sequencing of 17182 people without hematological abnormalities was analyzed. Changes in 160 genes (small insertions, deletions, and single nucleotide variants) that are involved in the development of hematologic cancers were investigated. The results of this work and several others [8,9,10] showed high and extremely varying mutation frequencies. Surprisingly, among all mutations, mutations of three genes are very common in different people: DNMT3A (DNA methyltransferase 3 alpha), TET2 (ten-eleven translocation 2), and ASXL1 (additional sex combs-like 1) genes. Less common mutations are JAK2 (Janus kinase 2), TP53, GNAS, PPM1D, and some other genes (Figure 1).

**Figure 2:**
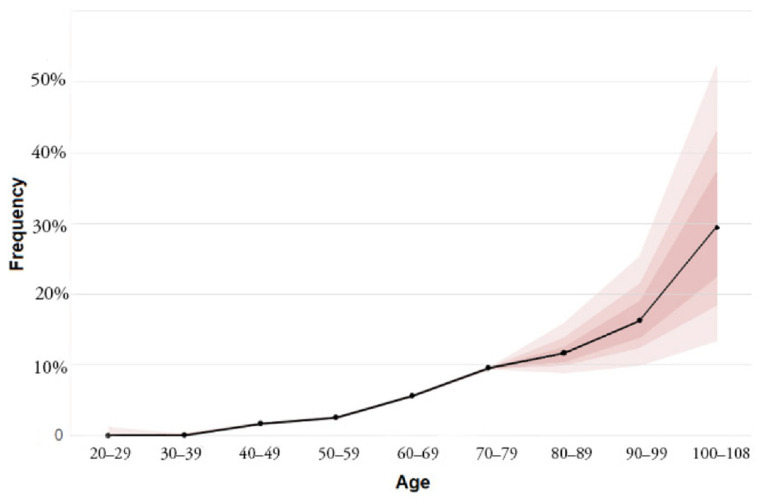
Increase in the frequency of mutations in genes implicated in hematologic cancers with age. Colored bands, in increasing lighter shades, represent the 50th, 75th, and 95th percentiles (modified from [11]).

It turned out that the frequency of these mutations increases with age. According to the same work [11], mutations in genes implicated in hematologic cancers under the age of 40 are very rare. Further, the frequencies increase steadily: after 60 years of life, it is 5.6%, after 70 years—9.5%, after 90 years, the frequency reaches 18.4% (Figure 2).

It is clear that these rates greatly exceed the incidence of clinically diagnosed hematologic cancer in the general population since a set of mutations is required for the formation of cancer. Although these people have an increased risk of developing blood cancer, real illness is very rare, and the condition has been named clonal hematopoiesis of indeterminate potential, or CHIP [12]. Subsequently, an alternative term appeared: age-related clonal hematopoiesis (ARCH) [13]. Long-term studies have shown the stability of such clones (at least 10 years) [14,15].

The detection rates of mutations depend on the type of analysis and the method of their detection [16]. In the paper by Zink et al. [17], investigators performed non-biased whole-genome sequencing without candidate driver mutations and increased the prevalence of mutations: from 0.5% in people under 35 years old to 50% or more in people over 85 years old. In research conducted by Young et al. [18], they used a special error correction method, due to which they found mutations in DNMT3A and TET2 in 95% of healthy 50–60-year-old individuals studied.

To estimate the prevalence of somatic mutations and changes in their frequencies, the development of new, more advanced technologies is required. However, it can already be argued that clonal hematopoiesis is very common already in middle age. Later in life, this phenomenon becomes almost ubiquitous [18].

At this stage of understanding, it is difficult to determine why clonal expansion occurs mainly only of particular mutations. It can be assumed that this process is influenced by both genetics and environmental factors, as well as changes in the bone marrow during aging [19,20]. One suggested explanation might be that if mutations lead to pro-inflammatory changes, such cells may have a survival advantage [21]. These questions require further study.

Anyway, the range of mutations leading to clonal hematopoiesis is much wider than the target genes involved in the study of carcinogenesis. An increased frequency of clonal hematopoiesis is found in cancer patients with different tumor localization [22,23,24]. This may be caused by the predisposition to carcinogenesis, as well as a reaction to chemotherapy (direct mutagenic effect of drugs and increased cell proliferation in response). The presence of clonal hematopoiesis in these cases worsens the prognosis also due to increased cardiovascular risks [25,26,27].

The term “therapy associated clonal hematopoiesis” has appeared. In this case, the frequency of mutations associated with resistance to therapy increases in cells, for example, tumor suppressor genes, such as TP53 and PPM1D [22,23,24,28,29]. Most intriguingly, clonal hematopoiesis is associated with a 40–50% increase in risk for all-cause mortality [8,11].

This is indicated by several investigations, including those with extensive coverage. Mosaicism was examined in 151,202 blood samples, and 8342 mosaic chromosomal alterations were found. These changes are more than the tenfold elevated risk of subsequent hematological cancer, and detectable mosaicism roughly doubled the risk for all-cause mortality (corrected for age, sex, and smoking status) [30].

The risk level is comparable to that of smoking [17]. The increased risk of all-cause mortality cannot be explained by the increased risk of hematologic neoplasms, which are relatively rare. What explanation can we offer? An association of CHIP with coronary heart disease and ischemic stroke was observed. Since atherosclerosis is the leading cause of death in the elderly, this explanation, in principle, passes.

## 3. Cells with Mutations Responsible for CHIP Are Often Characterized by Pro-Inflammatory Changes

### 3.1. DNMT3A

As noted above, changes in the DNMT3A gene are most often involved in clonal hematopoiesis in the elderly. This gene belongs to a family of cytosine methylases. They catalyze the addition of the methyl group to genomic DNA. Thus, the DNMT3A gene is involved in the epigenetic regulation of gene expression [31].

Many studies reveal that DNMT3A is involved in the regulation of inflammation at the level of cells—key participants (macrophages, T-lymphocytes, and mast cells). Through gene editing, it was shown that the loss of DNMT3A function in hematopoietic cells contributed to the development of heart failure by increasing inflammation. It has been shown that transplantation of cells with an altered DNMT3A gene results in an increased accumulation of macrophages in the heart of mice after angiotensin II administration. DNMT3A-deficient macrophages have an increased expression of pro-inflammatory cytokines and chemokines such as IL-6, CXCL1, CXCL2, and CCL5 following stimulation with LPS [32]. The DNMT3A gene has been shown to restrict the development of inflammation by suppressing the expression of IL-13 in T helper-2 cells [33].

It has been shown that the product of the DNMT3A gene restrains inflammatory pathways in mast cells [34]. The DNMT3A gene product is involved in the regulation of inflammation by influencing the polarization of T-cells [35,36]. Thus, it is clear that DNMT3A has many immunomodulatory properties with a predominance of anti-inflammatory activity. The mechanism of such properties is not fully clear, but they seem to be associated with the epigenetic regulation of transcription factors’ activity.

### 3.2. TET2

TET2 is one of the most common genes, mutations of which lead to an increased risk of death due to cardiovascular diseases. More than 130 TET2 mutations have been described. Most mutations result in loss of function [8,11,37].

The product of TET2 (ten-eleven translocation 2) converts 5-methylcytosine into 5-hydroxymethylcytosine—it is the first step in the cytosine demethylation process. Thus, TET2, as well as DNMT3A, is involved in the epigenetic regulation of gene expression.

Studies involving Tet2-deficient macrophages revealed they have an increased expression of pro-inflammatory cytokines after LPS stimulation. Increased levels of IL-1β, IL-6, and CCL5, but not CXCL1 and CXCL2. This distinguishes the expression profile from DNMT3A-deficient macrophages [32]. One possible reason for the clonality of TET2 mutations is that they cause an increased hematopoietic stem cell self-renewal. Moreover, in animals with TET2 deficiency, extramedullary hematopoiesis is observed, with an increase in monocytes and neutrophils within the spleen [38,39].

In experiments simulating the effect of TET2 mutations on the development of atherosclerosis, TET2–deficient hematopoietic cells were transplanted into *Ldlr*−/− mice prone to atherosclerosis [40]. The acceleration of the development of atherosclerosis was observed. Further, transplantation of cells with myeloid-specific ablation of Tet2 was sufficient for the effect. Probably it is the cells of myeloid origin; most likely, macrophages mediate the acceleration of atherosclerosis with TET2 mutations [40].

The heterozygosity for the TET2 mutation of the transplanted cells was sufficient to accelerate atherosclerosis, albeit to a lesser extent. In a similar article [10], it was also shown that Tet2-deficient macrophages accelerate the development of atherosclerosis and have pro-inflammatory properties, expressed in increased production of cytokines, including IL-1β. The authors suggest that the effect on atherosclerosis is mediated by the increased recruitment of monocytes to the affected area through increased CXCR2 signaling. It is known that the content of IL-1β is increased in the plaques of atherosclerotic mice.

A significant result of this work is that the authors revealed an increased level of inflammation in different tissues of mice transplanted with Tet2 knockout bone marrow. Inflammatory infiltrates in the lungs and liver, the development of prominent xanthomas in the spleen and middle ear, marked foam-cell accumulation, and glomerulosclerosis in the kidney were observed [10].

The mechanism of action of TET2 mutations may consist of the suppression of the transcriptional activation of pro-inflammatory genes by TET2 via recruiting histone deacetylase 2 to the gene promoter [41]. This may be true for both IL-1β and NLRP3 components [40].

### 3.3. JAK2

The JAK2 gene is also one of the most common variants of mutated genes in the hematopoietic system. JAK2 is a member of the Janus family of cytoplasmic non-receptor tyrosine kinases. The JAK-STAT signal transduction pathway is responsible for mediating signals of over fifty cytokines, growth factors, and hormones, including erythropoietin and thrombopoietin [42].

The most common JAK2 mutation is designated as JAK2V617F. It occurs in both myeloproliferative disorders and clonal hematopoiesis [43]. A blood test of 19,958 adult persons showed that 3.1% of individuals harbor this mutation [44]. Acceleration of murine heart failure was shown when transplanting bone marrow cells carrying the JAK2V617F mutation into mice [45]. At the same time, an increased level of pro-inflammatory mediators within the hearts was shown.

In a similar work, transplantation of bone marrow cells carrying the mutation JAK2V617F to *Ldlr*−/− atherosclerosis-prone mice led to the acceleration of the development of atherosclerosis [46].

JAK2V617F macrophages has an increased expression of pro-inflammatory cytokines, which activated inflammasomes, increased p38 MAPK signaling, and decreased activity of c-Mer tyrosine kinase, a key molecule mediating efferocytosis. It is worth noting that the authors noticed a high prevalence of incomplete erythrophagocytosis, which they associate with decreased expression of CD47 (a “don’t eat me” signal) on JAK2V617F erythrocytes.

Both human and murine neutrophils carrying JAK2V617F mutations have an increased ability to form NET. It is known to increase the risk of thrombosis [47].

The pro-inflammatory changes that accompany the JAK2V617F mutation served as the basis for the inclusion of anti-inflammatory drugs in the treatment protocols for patients with myeloproliferative diseases. Over the past decades, these have included corticosteroids, interferons, immunomodulatory imide drugs, and, more recently, JAK inhibitors [48].

## 4. Mitochondrial DNA Mutations and Their Impact on Proinflammatory Cellular Phenotype

The association of atherosclerosis with impairment of mitochondrial function was discovered at the level of clinical manifestations, oxidative stress, and other risk factors [49]. It was found that the impairment of mitochondrial function can be related to medial degeneration and arterial aging [50] because of changes in the expression of genes regulating the number of mitochondria. The correction of mitochondrial functional activity can delay the process of aging in the case of arterial vessels.

Several recent studies provide pieces of evidence supporting the connection between mtDNA mutations and atherosclerosis. These studies were conducted on arterial wall samples and leukocytes obtained from atherosclerosis patients. The majority of identified mutations are related to mitochondrial transfer RNA, mitochondrial ribosomes, and different mitochondrial-encoded respiratory complex subunits. It has been proposed that the presence of these mutations triggers mitochondrial dysfunction and, therefore, ROS production, which enhances the appearance of atherosclerotic plaques and increases the thickness of the intima and medial layers in carotid arteries [51].

Cancer is associated with impaired energy production in the cell and inflammation. The events leading to cancer development are often related to chronic inflammation and infection. Thus, mitochondria are crucially involved in cancer development, including the process of immune reaction. Unrestricted tumor development can be connected with the suppression of the process of immune response related to inflammation [52].

Cancer growth and impairments in mitochondrial functions may be caused by mtDNA mutations [53]. Dysregulation of energy production in the cell is associated with mitochondrial dysfunction and characterizes the process of oncogenesis [54,55]. Inflammation and cellular homeostasis can be affected by pathological changes in mitochondrial function during cancer [56,57,58]. An interesting observation was recently made by Smith et al. Their study revealed a contribution of mtDNA mutations to cancerogenesis and aging via an OXPHOS impairment. The authors linked the age-associated accumulation of mtDNA mutations to OXPHOS deficiency, which promotes metabolic remodeling. Consequently, it can functionally contribute to accelerated intestinal cancer development [59,60].

Asthma, cystic fibrosis, pulmonary fibrosis, pulmonary hypertension, and chronic obstructive pulmonary disease (COPD) are associated with dysfunction in mitochondria and dysregulated inflammation in the lungs [61,62,63]. More precisely, mutations in mitochondrial genes NLRX1 and MAVS were associated with COPD [64,65,66]. In addition to that, patients with this disease were shown to have a lower amount of mitochondria, a change in mitochondrial DNA content, impaired mitochondrial functions in skeletal muscles [67,68,69,70] and alveolar macrophages [71], mitochondrial fission associated with the degradation of lung tissue [72].

Autoimmune rheumatic diseases (ARD) are related to autoreactive self-inducing adaptive and innate immune reactions, which result in tissue damage. Such examples are systemic lupus erythematosus (SLE) and rheumatoid arthritis. Unregulated activation of innate and adaptive immune responses induces chronic inflammation and is essential for ARD development during every step [73]. Monocytes, macrophages, and their cytokines are the key components of autoimmune disease development [74].

Mitochondrial abnormalities can cause the release of mtDNA and stimulate the immune response, which is linked to various neurological diseases. mtDNA content and ccf-mtDNA in plasma and CSF have been analyzed in a number of these diseases. Among such diseases are major depressive disorder, Alzheimer’s disease, Parkinson’s disease, multiple sclerosis, schizophrenia, and others. The range of mutations causes accumulation of damage in different ways during the pathogenesis of neurodegenerative disorders. Moya et al. propose that accumulation of defective mitochondria due to impaired mitophagy and the stimulation of oxidative stress are common factors that link mtDNA-dependent inflammation within these pathologies [75].

The evidence was found that impairments in mitochondrial function can stimulate human synoviocytes’ inflammatory response with its modulation in the direction of the greater increase due to the induction by IL-1β [76]. The work of Caric et al. provides pieces of evidence that in iNOS, BCL-2 and MMP-9 are involved in the regulation of hip osteoarthritis [77].

An interesting observation was made that most of the identified mtDNA mutations are mild or non-pathogenic, so they may not be a cause of a certain pathogenic phenotype development. Recently, some investigations have revealed multiple mtDNA mutations linked to hypertension, which allows the suggestion of its maternal transmission. Thus, aberration of the tRNA levels causes the decreased rate of mitochondrial protein synthesis and a reduction in mitochondrial protein levels in the mutant cells, altered complex I/III activity, electron leakage, and enhanced ROS production. Subsequently, a damaged mitochondrial respiratory chain caused a vicious cycle: increased ROS production means a higher rate of mtDNA mutations and cell death. Thus, MtDNA mutations and deletions contribute to oxidative stress and mitochondrial dysfunction, which may be involved in the development and pathogenesis of CVD, in particular, hypertension and atherosclerosis [78].

The variety of human diseases associated with inflammation is also characterized by excessive ROS production. It is not a surprise that mitochondrial dysfunction is also involved in human diseases with underlying inflammatory pathologies, such as diabetes mellitus and cardiac dysfunction [79].

The increase in the level of mitochondrial ROS can contribute to cell death caused by apoptosis and lymphopenia, and increased inflammation can be caused by necrosis of SLE lymphocytes [80].

The mechanism of pathogenicity in ARD may be related to the impaired removal of apoptotic cells by macrophages leading to the accumulation of apoptotic cell-related autoantigens, including oxidized proteins [80]. Apoptotic cells, which were not removed by macrophages, experience secondary necrosis, membrane disruption, and intracellular components, including proteins, get released in the extracellular environment causing inflammation due to the induction of autoreactive B- and T-cells [81].

Recent evidence suggests that Parkinson’s disease is also related to impaired mitophagy, which leads to the release of mitochondrial DNA (mtDNA), which, in turn, stimulates inflammation. Borsche et al. revealed that in individuals carrying mutations in PRKN/PINK1, IL6 and circulating cell-free mtDNA levels can serve as markers of Parkinson’s disease state and progression, respectively. This study demonstrates an essential role of inflammation in Parkinson’s disease pathogenesis and, what is more, links this process to mitochondrial function abnormalities [82]. Another study, performed by Sliter et al., support these findings. They observed a strong inflammatory phenotype in both Prkn−/− and Pink1−/− mice following exhaustive exercise and in Prkn−/− mutator mice, which accumulate mutations in mitochondrial DNA (mtDNA). Inflammation resulting from exhaustive exercise or mtDNA mutation can be rescued completely by the concurrent loss of STING, a central regulator of the type I interferon response to cytosolic DNA. The loss of dopaminergic neurons from the substantia nigra pars compacta and the motor defect observed in aged Prkn−/− mutator mice are also rescued by loss of STING, suggesting that inflammation facilitates this phenotype. Humans with mono- and biallelic PRKN mutations also display elevated cytokines. These results support a role for PINK1- and parkin-mediated mitophagy in restraining innate immunity disease. Therefore, the authors hypothesize that parkin and PINK1 prevent inflammation and neurodegeneration by clearing damaged mitochondria, thereby preventing increases in cytosolic and circulating mtDNA, suggesting a new model for how mitophagy may mitigate Parkinson’s disease [83].

Due to the function of mitochondria as a power plant of the cell, mtDNA is more susceptible to the damage caused by oxidation than DNA localized in the nucleus [84]. The impairments of mtDNA can lead to defects in mitochondrial functions, which, in turn, can generate more ROS causing repeated cycles of mtDNA damage. When the pressure of oxidative stress is moderate, the mitochondrial machinery still can be recovered upon the increase of copy number of mtDNA as compensation for the damaged one. However, the further increase in ROS production can cause a decrease in mtDNA copy number and impairment in mitochondrial activity [85].

One of the factors of aging is related to mitochondrial dysfunctions, changes in the amounts of mitochondria in the cell, etc. [86]. The term inflammaging refers to the idea that aging causes the formation of the pro-inflammatory situation in the organism [86], leading to pathological (chronic) inflammation-causing mortality and decreasing the quality of life in an older part of the population [87,88,89].

It is known that different processes involving mitochondria (oxidative phosphorylation, mitophagy, etc.) become impaired during aging [90,91]. In addition to that, defects in mtDNA reparation cause the impairment of mitochondrial function and, as a result, accelerate aging [92]. Dysfunctions of mitochondria happening as the organism gets older eventually result in mitochondria-associated damage-associated molecular patterns (DAMPs) being released and induction of innate immunity, and the development of age-associated chronic diseases.

An interesting association between MACE (major adverse cardiovascular event) and mtDNA4977 deletion was observed in the study by Vecoli et al. Short leukocyte telomere length and high mtDNA4977 deletion showed independent and joint predictive value on adverse cardiovascular outcomes and all-cause mortality in patients with CAD. These findings strongly support the importance of evaluating biomarkers of physiological/biological age, which can predict disease risk and mortality more accurately than chronological age [93].

Experiments with mice carrying mutations in the mitochondrial polymerase gene have produced remarkable results. These mice are referred PolgA, and in their mitochondria, mitochondrial mutations accumulate during their lifetime. PolgA mice age rapidly, starting from 6–8 months of age, and have significantly reduced longevity. They have various pathologies associated with aging, including hair graying, alopecia, osteoporosis, hemopoietic stem cell decline, cardiomyopathy, kyphosis, and frailty. The mouse phenotype became extremely inflammatory. Contrary to expectations, no signs of ROS increase were found in the tissues of these mice [94,95,96].

It is possible that mitochondrial mutations still lead to an increase in ROS since antioxidants improve the state of PolgA mice [97]. There is a possibility that cells with mitochondrial mutations leading to an increase in ROS may be eliminated during development, leading to accelerated senescence of progenitor cells [97]. An increase in ROS damages telomeres and increases senescence accordingly. On the other hand, an increase in ROS may just lead to apoptosis, which increases the turnover of progenitors, and so on.

The interpretation of experiments with “mutator” mice maybe that mitochondrial mutations generally induce a pro-inflammatory response and trigger inflammation. Given the significant difference in the lifespan of humans and mice, it can be assumed that mitochondrial mutations are of greater importance for humans since the number of mitochondrial DNA replications (and, accordingly, the number of replication errors) in humans should be significantly higher.

At least, several possibilities can be envisioned as to how mitochondrial mutations contribute to inflammation:

1. Mitochondrial mutations damage mitochondria. This can lead to their destruction and facilitate the release of mitochondria-derived alarmins, including mtDNA.
2. Mitochondrial mutations are capable of weakening the energy potential of mitochondria, leading to increased glycolysis. In the case of monocytes/macrophages, this may contribute to the polarization in M1 [98,99].
3. Mitochondrial mutations can lead to an increase in ROS, which causes cellular damage, leading to aging or cell death and contributing to the appearance of an aging-associated pro-inflammatory phenotype (SASP).
4. Disruption of mitophagy and the corresponding release of mtDNA into and out of cells. The circulating cell-free mitochondrial DNA appears in blood plasma [82,100].

We should note that all the described mechanisms lead to the circulation of mitochondrial alarmins throughout the body and also contribute to (possibly) reprogramming of macrophages towards M1. From this, it can be concluded that an additional mechanism of aging associated with blood cells spreads increased inflammation throughout the body, contributing to the development of multiple pathologies associated with aging, i.e., increased comorbidity.

## 5. Mutations in Hematopoietic Cells Increase Inflammation at the Body Level, Thereby Accelerating Aging

As shown above, the most common nuclear mutations in hematopoietic cells change their phenotype towards increased inflammation. Mitochondrial mutations have a similar effect.

Leukocytes are directly involved in the processes of inflammation. These cells migrate to the area of inflammation from the circulation and accumulate there. If these cells have pro-inflammatory changes, then the existing focus of inflammation is less likely to go into the resolving phase. On the contrary, pro-inflammatory cells should intensify the process, and inflammation will continue or even spread further. For example, if cells have mitochondrial mutations leading to mitochondrial destabilization, then such cells will carry out their functions to fight infection, regardless of time, since the breakdown products of mitochondria are similar to bacterial.

Interesting observations were made in the last years considering the Ercc1-deficient or Ercc1-knockout mouse model. The Ercc1 gene encodes a crucial DNA repair protein, Excision repair cross-complementing group 1. These mice accumulate spontaneous, oxidative DNA damage of the same kind as the wild-type (WT) mice, but at a faster rate. Moreover, many features of natural murine aging, as well as human aging, are present in Ercc1-/Δ mice. Emerging use of these mice allows studying age-related signaling pathways, including identifying different types of senescent cells and their key senescent cell anti-apoptotic pathways (SCAPs). The most important use of this model is the evaluation in vivo of senolytic drugs and other gerotherapeutics [101]. A study by Robinson et al. revealed the oxidative stress origin of Ercc1-/Δ-associated accelerated mutation accumulation. Their findings state that nuclear genotoxic stress arises, at least in part, because of mitochondrial-derived ROS, and this spontaneous DNA damage is sufficient to drive increased levels of ROS, cellular senescence, and the consequent age-related physiological decline [102]. Another study conducted by Yosefzadeh et al. demonstrated that Vav-iCre+/−; Ercc1−/fl mice were healthy into adulthood, then displayed premature onset of immunosenescence. This process was accompanied by the attrition and senescence of specific immune cell populations, along with impaired immune function, similar to changes that occur during aging in wild-type mice. Another important observation is that non-lymphoid organs also exhibited enhanced senescence and damage, which suggests that senescent, aged immune cells can promote systemic aging [103].

About 20 years ago, the term “inflammaging” appeared [87,88]. This term combines aging and inflammation and explains the relationship between chronic inflammation and the development of aging-associated pathologies. It is known that aging in humans is closely associated with sluggish, chronic inflammation [104,105]. It is believed that the sources of such chronic inflammation can be senescent cells accumulating with age, possessing SASP, various cellular debris, fragments of the extracellular matrix, etc. It has been shown that many indicators of inflammation circulating in the blood are strong predictors of age-related morbidity and mortality [106,107].

The position of inflammaging theory has been greatly strengthened in recent years with the development of senolytics. Selective elimination of senescent cells from the body caused not just a decrease in inflammation and an improvement in the physical functions of many systems of the body but also increased life expectancy [108,109,110]. It finally became clear that chronic inflammation is one of the mechanisms of aging.

The theory of aging based on the accumulation of somatic mutations was proposed more than 50 years ago [111]. It explained in general terms that the accidental accumulation of errors leads to a catastrophe of errors, expressed in a decrease in the optimal functioning of all systems.

In our case, the accidental accumulation of mutations in cells involved in the executive mechanisms of inflammation prevents the effective (developed as a result of natural selection) work of inflammation mechanisms to restore the lost status quo. This is a more significant violation. On the other hand, the rate of occurrence and spread (formation of clones) of mutations is higher in rapidly proliferating tissues (bone marrow), which also increases the significance of such mutations [7].

A centenarian (105+) can be considered as a person with delayed aging or with the characteristics associated with healthy aging [112]. The involvement of bone marrow cell mutations (clonal hematopoiesis) in the process of such healthy aging has recently received very significant confirmation. The study investigated somatic mutations in very old people [113]. The work showed that people who have reached the age of 105+/110+ are distinguished by the increased efficiency of DNA repair mechanisms, leading to a decrease in the number of somatic mutations. They have a lower mutation load than younger, healthy people.

Analysis of 7 genes involved in clonal hematopoiesis (DNMT3A, TP53, ASXL1, TET2, SF3B1, PPM1D, JAK2) showed that, in addition to the JAK2 gene, all other genes in very old people mutate less often than the same genes in controls with an average age of 68 years. At the same time, the differences for two separate genes are more than two times (DNMT3A, ASXL1) and are quite significant.

It is possible that in cases of very old age, control over inflammation becomes a more important factor of successful longevity, ensuring not only survival but the preservation of capability and cognition [114].

## 6. Conclusions and Prospects

**Figure 3:**
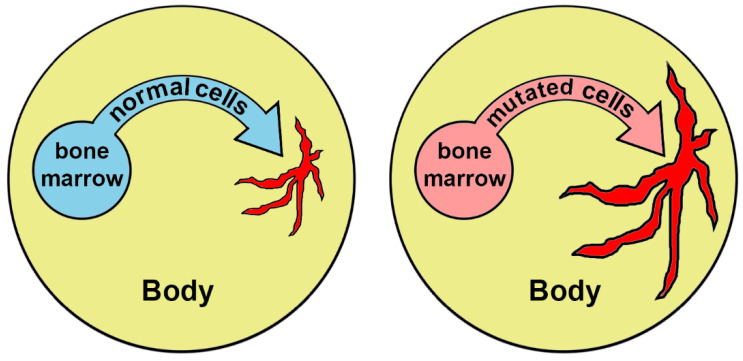
Extension and chronification of inflammation under the influence of hematopoietic cells carrying mutations. Areas of inflammation are marked in red.

The mechanism of aging considered in this review (Figure 3) should be taken into account as an important factor of the aging process:

1. It begins to work and strengthens its activities in the process of life. This mechanism is activated as mutations accumulate. At the same time, starting from middle age, its effect is enhanced. If a person has hereditary mutations, the mechanism can work from the very beginning of his/her life.
2. The action of the mechanism should enhance comorbidity. Since cells from the bone marrow participate in inflammatory processes throughout the body and modify them, inflammation is generalized and encompasses all organs and tissues. As a result, there is a “synchronization” of inflammation in all organs and tissues, all systems become decrepit, and the likelihood of a fatal outcome increases.
3. Knowledge of this mechanism allows us to hope for the possibility of counteracting it. These interventions can be quite universal and not unique for each patient due to the effect of CHIP.
4. Immediately, we would like to offer a universal treatment—bone marrow transplantation from young donors. This idea, of course, should work, but for various reasons, it cannot be massively implemented.
5. Approximately 100 years ago, the idea of blood transfusion from young donors for rejuvenation was developed in the USSR by the director of the world’s first blood transfusion institute, Alexander Bogdanov (Malinovsky). He died in 1928 after regular blood transfusion as a result of the then-unknown Rh conflict. The effect of blood transfusion can likely be quite long since it is not limited by the time of survival of transfused cells in the blood but, possibly, will contribute to the resolution of some of the sluggish inflammatory processes. If this scenario is realized, then the bone marrow’s cells will no longer support these areas of chronic inflammation. Interestingly, recent findings contribute to the beneficial effect of neutral blood exchange (NBE) that resets the signaling environment to a pro-regenerative state via dilution of old plasma. Comparative proteomic analysis performed by Mehdipour et al. on serum from NBE, and a similar human clinical procedure of therapeutic plasma exchange (TPE), showed a molecular re-setting of the systemic signaling milieu, interestingly, elevating the levels of some proteins, which broadly coordinate tissue maintenance and repair and promote immune responses. Investigators state that significant dilution of autoregulatory proteins that crosstalk to multiple signaling pathways (with their feedback loops) would, through changes in gene expression, have long-lasting molecular and functional effects. Their further work admitted the rejuvenating effect of NBE on the mouse brain [115,116].
6. Of course, the ideas of genomic editing of bone marrow stem cells look more realistic nowadays. But these technologies are not sufficiently developed in our time and are associated with health risks.
7. Given the practice, aging people can be recommended with time-tested and well-tolerated anti-inflammatory drugs (for example, aspirin) for continuous use.
8. A promising target may be circulating monocytes because they are key players in the regulation of inflammation. A decrease in the proinflammatory status of monocytes should reduce the level of most current inflammatory processes in the body and help resolve some of the foci of chronic inflammation.

## Data Availability

Not applicable.
